# Combined Factors for Predicting Cognitive Impairment in Elderly Population Aged 75 Years and Older: From a Behavioral Perspective

**DOI:** 10.3389/fpsyg.2020.02217

**Published:** 2020-09-10

**Authors:** Zhixiong Yan, Xia Zou, Xiaohui Hou

**Affiliations:** ^1^Psychology Department, Nanning Normal University, Nanning, China; ^2^Guangxi College for Preschool Education, Nanning, China; ^3^Faculty of Industrial Education and Technology, King Mongkut’s Institute of Technology Ladkrabang, Bangkok, Thailand

**Keywords:** cognitive impairment, protective/risk factors, elderly population, lifestyle, logistic regression

## Abstract

To unravel the combined effect of risk and protective factors that may contribute to preserve or impair cognitive status, this prospective cohort study systematically investigated a cluster of factors in elders aged 75 years and older from Guangxi Longitudinal Cohort (GLC) dataset. GLC has tracked 630 oldest-elders for two times within 2 years and will continue to follow two times in the next 4 years. At baseline geriatric assessment, sociodemographic information (e.g., education, Mandarin, marriage, and income), physical status [body mass index (BMI), chronic disease/medicine], lifestyle factors (smoking, alcohol, and exercise), and self-rated mental health (self-care, well-being, anxiety) were recorded by online interview. With 2 years’ follow-up, Mini-Mental State Examination (MMSE) and memory test were performed through person-to-person interview. The performance of MMSE was applied to represent the responder’s cognitive status which classified into cognitive impairment and normal group based on a cutoff point of 20. An age-related cognitive declining trend of 15 stratified factors was observed, though with a small effect size (R-square: 0.001–0.15). The odds of exposure or non-exposure on factors (memory, self-care, exercise, income, education, and literacy) had a significantly different effect on cognitive impairment through multivariate analysis after adjusting other confounding variables. Through stepwise multiple logistic regression analysis, the following 12 factors/index would be integrated to predict cognitive impairment: gender, physical health factors (BMI, chronic disease), socioeconomic and lifestyle factors (education, literacy, Mandarin, marriage, income, and exercise), and psychological health factors (memory, self-care cognition, and anxiety). Related clinical and nursing applications were also discussed.

## Introduction

Cognitive impairment, such as deterioration in memory, attention, and language, was considered as an inevitable trend of aging experienced by majority of elderly people (Folstein et al., 1985), the extent of which is strongly affected by individual variables (e.g., lifestyle, socioeconomic status) and their interactions. For someone, even the detail of experienced things could clearly be recalled, while some peers could not remember their own name. In current decades, the prevalence of cognitive impairment (e.g., mild cognitive impairment and dementia) has increased dramatically (Boyle et al., 2006) and considerable interest was attached in science research community (Deary et al., 2009). Taking China as an example, the estimated annual expenditures for cognitive impairment in the elderly population are predicted to be US$69 billion in 2020, which stressed a detrimental effect on family and other carers (Xu et al., 2017). As chronic and complicated characteristics, the effect of medical intervention to modify the course of cognitive impairment has not been effective and even hard to clearly attenuate impairment progression (Rocca et al., 2011). For cognitive impairment may be an agent of lifestyle-based causes, potentially modifiable behavioral factors are alternative to delay the onset of cognitive impairment (Friedman et al., 2015).

Accumulating epidemiological evidence indicates that psychological, environmental, and social factors can help to alleviate cognitive impairment and improve cognitive preservation (Wang et al., 2006; Rocca et al., 2011; Roberts et al., 2015; Arnau et al., 2016; Clare et al., 2017; Klimova et al., 2017; Lamblin et al., 2017; Zhang et al., 2017). A healthy lifestyle (refraining from smoking, moderate alcohol consumption, more physical activity/cardiorespiratory fitness, a Mediterranean-style diet, and more social and mentally stimulating activity) was associated with better cognitive performance and resilience (Hughes and Ganguli, 2009; Fung et al., 2011; Bielak et al., 2014; Dardiotis et al., 2014; Satizabal et al., 2016; Bubbico et al., 2019). Socioeconomic adversities (e.g., illiteracy, poor occupational achievement, and low income) could be potentially attributed to dementia (Scazufca et al., 2010). However, the measurements of these studies were heterogeneous with cross-sectional design and partial epidemiological factors. Comprehensive factors assessment and prediction were still lacking. Few studies have systematically examined the odds of the exposure and non-exposure of lifestyle factors. Moreover, exploring factors in a comprehensive and aggregated way would be a promising direction for preserving cognitive capacity. Thus, a systematic exploration of these factors is needed, and aggregately considering them was emphasized in current research. To collect factors in multiple level and as rich as possible, we classified the factors in lifestyle, socioeconomic, psychological, and physical aspect. Several pivotal modifiable factors associated with cognitive impairment will be refined after exploring the effect of each factor. The application for elder people and clinicians was suggested.

Besides, the diagnostic methods such as mild cognitive impairment (MCI) measurement were time-consuming and impractical for aging community survey (Rohrer et al., 2005). As a valid and brief tool of mental state assessment, Mini-Mental State Examination (MMSE, 30 items as well as 5–10 min testing time) covered a variety of cognitive competencies including orientation, memory, attention, reading, and writing with good identification property (Folstein et al., 1975). Here, the current study applied MMSE as a primary tool to measure cognitive impairment of elder individuals aged 75 years above. The aim of this study is to conduct a systematic analysis of factors and explore which is the best combination of protective and risk factors of cognitive impairment.

## Materials and Methods

### Subjects

**FIGURE 1:**
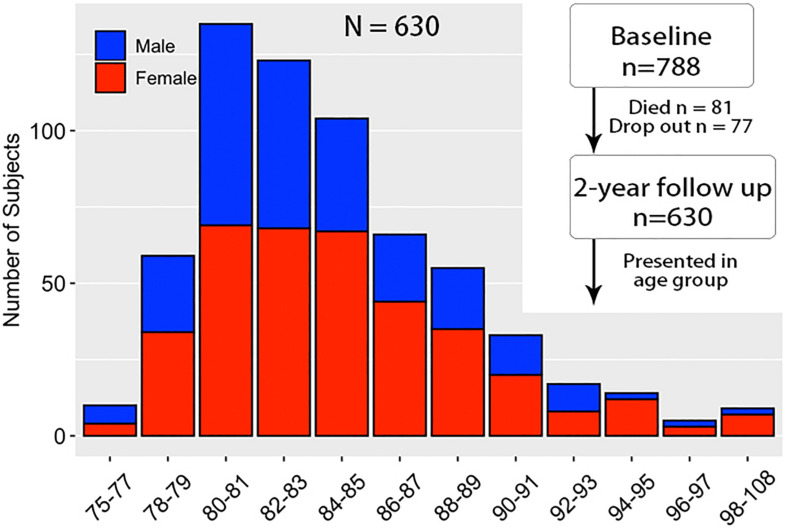
Subjects age-group distribution and collection flowchart.

A total of 788 subjects with normal cognitive function aged 75 years and above were randomly collected at baseline based on census track in 13 communities according to Guangxi Longevity Cohort Project (GLCP). After 2 years’ interval, the final sample consisted of 630 subjects (259 males, 371 females, mean age: 84.23) through interview in person (Figure 1). Subjects who died (81), not reached (30), and disconnected (47) were excluded from the following data analysis. The following participation rate was 80%.

### Factors and Stratified Criteria

At baseline, multidimensional factors were collected in sociodemographic characteristics (gender, the number of children, marriage, income, education, and Mandarin), lifestyle factors (smoking, alcohol, and exercise), physical status [body mass index (BMI), eyesight, chronic disease/medicine], and self-rated mental health factors (self-care, well-being, and anxiety) through telephone interview. Subjects were asked questions such as: How do you think your well-being? Are you a smoker? How many cigarettes do you smoke per day? The interviewer recorded the answer in specific points based on the subject’s response. All variables were stratified into two or four levels.

#### Sociodemographic Characteristics

Sociodemographic information was available regarding gender, age, up-bring children, education, Mandarin, and income. The number of children was divided as three children or more and less than three children. Marriage was classified as married and unmarried (including single, divorced, or widowed). Income grouped by more than 500 RMB and less than 500 RMB. Education was grouped as uneducated (never received education), less than 5 years, and more than 5 years. Mandarin was classified as capable (understanding and speaking Mandarin) and unable (cannot understand and speak Mandarin) groups.

#### Physical Health Factors

A brief measure of physical functioning was based on three separate tests of physical ability, regarding BMI (subject’s weight in kilograms divided by the square of height in meters), eyesight, and chronic disease/medicine condition. Subjects whose BMI < 18.5 were taken as underweight, > 24 as overweight and range between 18.5 and 24 as normal. Eyesight was divided by normal (eyesight test < 1.0) and abnormal (eyesight test > 1.0) groups. Chronic disease/Medicine had two levels (taking, none) according to having chronic disease (like cardiovascular disease, respiratory disease) and taking medicines or not.

#### Lifestyle Factors

Smoking was classified as no smoking and smoking, whereas alcohol consumption was grouped into alcohol consumption and no alcohol consumption. Exercise was classified as > 50 min per day and < 50 min per day.

#### Self-Rated Mental Health

Self-rated mental health factors (anxiety, well-being, and self-care) were recorded by a single-item question with five alternative choices regarding excellent (receiving 5 points), very good (receiving 4 points), good (receiving 3 points), fair (receiving 2 points), and poor (receiving 1 point). Each specific component (like anxiety) was then divided into three stratified levels as low level (1–2 points), moderate level (3 points), and high level (4–5 points).

### Follow-Up Assessment

With 2-year follow-up, the evaluation including two tests was conducted by coordinator interviewer in person. One test was MMSE (Molloy, 2014) as an index of cognitive status in which subjects are assigned into two groups, the cutoff point for the MMSE performance was 20. MMSE was commonly used to distinguish subjects into with and without cognitive impairment (Uffelen et al., 2008). MMSE score above 20 grouped into normal group, whereas score below 20 was treated as cognitive impairment group.

To validate the cognitive measurement, digit span test was utilized as supplemental cognitive measurement in addition to protective risk in which included 17 items (sequential memory: nine items; reversed order memory: eight items). Each item had 1 point if a subject gave a correct answer. According to the performance of the digit span task, memory was classified as four groups according to the sum of points: excellent (> 11.5), good (> 7.86), medial (≥ 4.2), weak (< 4.2).

In the spirit of collaboration and open science, the data are available for application and can be freely accessed at data sharing part in our lab web page: http://yanlab.club/index.php/info/128.html.

### Statistical Analyses

**FIGURE 2:**
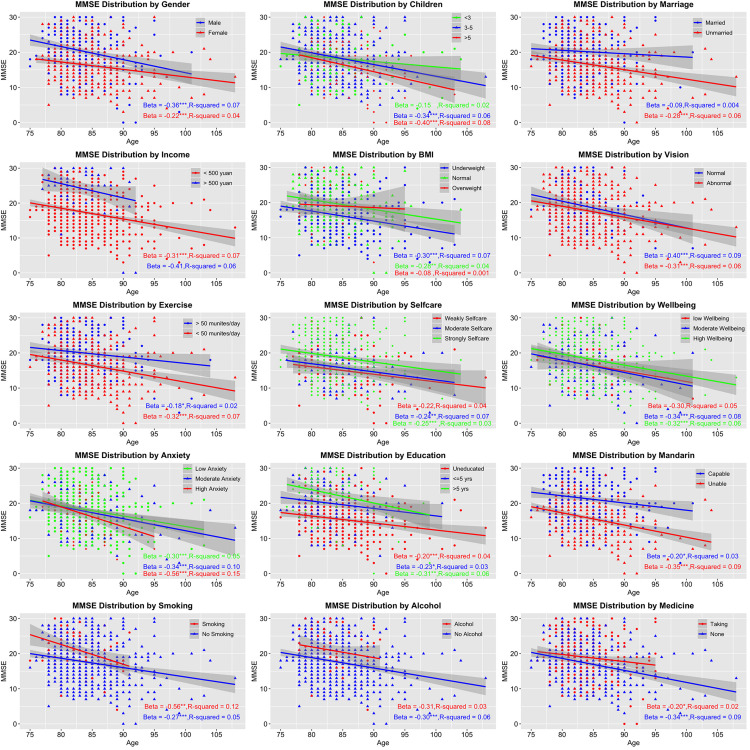
Age-related trends of cognitive changes by stratified factors. The vertical axis presents Mini-Mental State Examination (MMSE) score symbolizing cognitive statue; the horizontal axis presents age. Beta (the strength of association) and R-square (effect size) of each stratified factor were also calculated. *p < 0.05; **p < 0.01; ***p < 0.001.

All statistical analyses were performed in *Epicalc* (Chongsuvivatwong, 2007), an epidemiological data analysis tool in R. The data with missing records were omitted before statistical analyses. The function *lm* based on the least squares method was used to perform age-related linear modeling for each factor. The attributes of β (coefficients of the independent variables) and R-square (effect size) were calculated (Figure 2). Multivariate analyses using logistic regression models (*glm*) were conducted to identify the effect of exposure and non-exposure in specific factors on cognitive impairment in which crude odds ratio (OR) and adjusted OR (adjusted for other variables) were conducted (Table 1). Stepwise logistic regression (*step*) was followed for removing non-significant independent variables according to Akaike’s Information Criterion (AIC) in which the optimal model with the lowest AIC value showed high likelihood or best fit. Specifically, the step removes each independent variable and compares the degrees of freedom reduced, the new deviance, and the new AIC. The results are increasingly sorted by AIC. The top one having the lowest AIC is the best one (Step 6 in Table 2).

**TABLE 1:** The exposure and non-exposure effects of risk factors on cognitive impairment.

	**Factors**	**Crude OR**	**95% CI**	**Adj. OR**	**95% CI**	***p* (Wald’s test)**
Age	75	1.00				
	75 + 1	1.09	1.05–1.14	1.01	0.95–1.07	0.78
Gender	Male	1.00				
	Female	3.74	2.58–5.41	1.58	0.85–2.94	0.15
Children	0–2	1.00				
	> 3	1.01	0.66–1.55	1.11	0.58–2.10	0.75
BMI	Normal	1.00				
	Low-weight (< 18.5)	3.07	2.08–4.53	1.56	0.91–2.68	0.11
	Over-weight (> 24)	1.20	0.66–2.21	1.05	0.40–2.74	0.92
Self-care	Medial	1.00				
	Weak	0.63	0.28–1.44	0.22	0.07–0.70	0.01*
	Strong	0.23	0.11–0.43	0.21	0.09–0.50	<0.001***
Well-being	Medial	1.00				
	Weak	1.42	0.58–3.51	1.79	0.52–6.15	0.35
	Strong	0.74	0.46–1.18	1.26	0.63–2.52	0.52
Anxiety	Medial	1.00				
	Weak	1.02	0.64–1.65	1.76	0.89–3.49	0.10
	Strong	1.35	0.63–2.88	1.64	0.58–4.64	0.35
Education	None	1.00				
	1–5 years	0.21	0.13–0.32	0.77	0.38–1.55	0.46
	> 5 years	0.08	0.05–0.13	0.34	0.14–0.83	0.02*
Literacy	Literate	1.00				
	Illiterate	9.59	6.33–14.52	2.92	1.41–6.05	0.004**
Mandarin	Able	1.00				
	Unable	5.17	3.49–7.54	1.45	0.79–2.68	0.23
Marriage	Married	1.00				
	Unmarried	3.02	2.10–4.36	1.35	0.77–2.35	0.30
Income	Low (< 500 yuan)	1.00				
	High (> 500 yuan)	0.10	0.05–0.20	0.32	0.12–0.84	0.02*
Smoking	Smoking	1.00				
	Non-smoking	2.54	1.52–4.26	0.90	0.41–1.96	0.79
Alcohol	Drinking	1.00				
	Non-drinking	2.58	1.51–4.41	1.19	0.55–2.56	0.65
Chronic disease/	Taking	1.00				
Medicine	Never	1.51	1.06–2.15	1.26	0.74–2.13	0.39
Exercise	High (> 50)	1.00				
	Low (< 50)	2.50	1.74–3.58	3.10	1.80–5.31	<0.001***
Memory	Excellent	1.00				
	Good	6.14	3.27–11.56	4.84	2.25–10.39	<0.001***
	Medial	24.98	11.90–52.46	12.21	4.90–30.42	<0.001***
	Weak	55.05	21.00–144.30	36.78	11.62–136.44	<0.001***

*BMI, body mass index; CI, confidence interval. ^∗^p < 0.05, ^∗∗^p < 0.01, ^∗∗∗^p < 0.001.*

**TABLE 2:** Crucial factors predicting cognitive impairment by stepwise regression.

	**Step 1**	**Step 2**	**Step 3**	**Step 4**	**Step 5**	**Step 6**
Age	+	+				
Gender	+	+	+	+	+	+
Children	+	+	+	+	+	
BMI	+	+	+	+	+	+
Self-care	+	+	+	+	+	+
Well-being	+					
Anxiety	+	+	+	+	+	+
Edu	+	+	+	+	+	+
Literacy	+	+	+	+	+	+
Mandarin	+	+	+	+	+	+
Marriage	+	+	+	+	+	+
Income	+	+	+	+	+	+
Smoke	+	+	+			
Alcohol	+	+	+	+		
Chronic disease/Medicine	+	+	+	+	+	+
Exercise	+	+	+	+	+	+
Memory	+	+	+	+	+	+
AIC	457.08	454.04	452.12	450.22	448.36	446.58

*BMI, body mass index; Edu, received education years; AIC, Akaike’s Information Criterion, in which a low value shows a high likelihood or a good fit. Each column shows an alternative model, the model of Step 6 was found as the optimal model with the lowest AIC.*

## Results

### Age-Related Trajectories of Cognitive Changes via Stratified Factors

A linear declining trend was found in all factors stratified in two to four levels (Figure 2). Several factors showed stronger decreasing tendency such as high anxiety (β = -0.56) and smoking (β = -0.56), while others like married condition (β = -0.09) and overweight (β = -0.08) showed weaker associations. Unfortunately, all associations only reached small effect sizes (*R*^2^ from 0.001 to 0.15).

### The Effects of Exposure and Non-exposure in Specific Stratified Factors

In multivariate regression analysis on which the effects of exposure and non-exposure were checked (Table 1), the most significant factor that contributed to cognitive impairment was memory. Compared to excellent memory subjects, five times increase in the odds of cognitive impairment, 12.21 times and 36.78 times for medial and weak memory subjects, respectively. Another significant mental health factor was self-care. Compared to medial level, weak level and strong level had only 0.22 and 0.21 times to developing cognitive impairment. Similarly, there are three sociodemographic factors that played as protective factors. As to non-education, subjects who received 1–5 years of education had only 0.77 times possibility resulting in cognitive impairment. If receiving education > 5 years, the odds decreased to 0.34 times. Similar to education, illiterate subjects had 2.92 times suffering cognitive impairment compared to literate subjects. Income is a representative index for subject’s economic status, by which high-income subjects (> 500 yuan per month) had just 0.32 times led to cognitive abnormal referenced to low-income ones (< 500 yuan per month). Among lifestyle factors, the influence of physical exercise < 50 min/day had 3.1 times odds to lead to cognitive impairment compared to exercise > 50 min/day. Other factors did not show pronounced difference between exposure and non-exposure.

### The Aggregated Factors That Best Predict Cognitive Impairment

**FIGURE 3:**
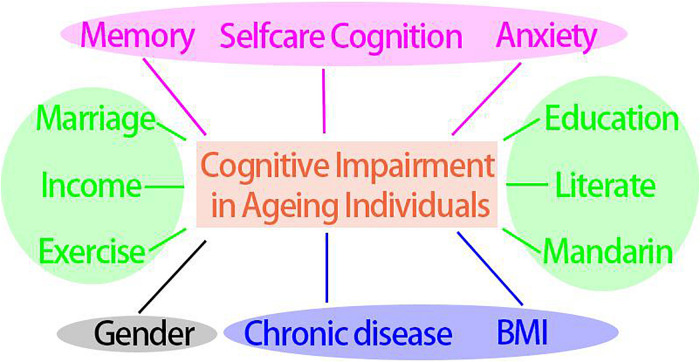
Key potential modifiable factors that contribute to cognitive impairment in aging individuals. Blue ellipse: physical health factors, pink ellipse: psychological factors, green ellipse: socioeconomic and lifestyle factors, blank ellipse: generally unmodifiable factor.

To check whether some crucial factors could combine to predict cognitive impairment as the optimal model, stepwise regression analysis was conducted (Figure 3 and Table 2). The Step 6 was taken as the optimal model for which its lowest AIC value showed the best fitting results (466.58). The model encompassed Gender, BMI, Self-care cognition, Anxiety, Education, Literacy, Mandarin, Marriage, Income, Chronic disease/Medicine, Exercise, and Memory. Those factors may be more effective when they are combined to predict and intervene cognitive impairment for elderly populations aged 75 and older.

## Discussion

As lifestyle and other factors played an enduring and interacted way in the cognitive aging process, uncovering the protective or risk effect on cognitive impairment was a great challenge. Though numerous findings indicated that lifestyle and other sociodemographic factors impacted on cognitive performance (Mensink et al., 1997; Walsh, 2011; Roberts et al., 2015; Zhang et al., 2017; Clare et al., 2017), it is hard to explore the precise mechanism of each factor on cognitive change. Taking the determinants together would be a promising alternative to comprehensively consider protective or risk factors.

In the present study, a similar age-related decreasing trend of cognitive status was found in each stratified factor. Each factor independently contributed to cognitive impairment but with a limited effect size (Deary et al., 2009). Besides, a pronounced exposure effect was found in the following risks *via* multivariate regression analysis: lifestyle factor (physical activity/exercise), socioeconomic factors (education, literacy, income), psychological health factor (self-care cognition), and memory. Exposure in these factors made dramatically different chances to be onset of cognitive impairment. Taking memory as an illustration, the risks of cognitive impairment increased more than 36 times among weak memory individuals compared to that of excellent memory ones. It would be reasonable to infer that working memory stimulation tasks may be one of the most beneficial approaches for preserving and improving cognitive capacity in elder adults. A working memory intervention study also suggested that memory training was an effective way for maintaining normal cognitive function (Heinzel et al., 2014). As for other factors in the current study, low-level exercise (<50 min/day) responders had 3.1 times odds to suffer from cognitive impairment in comparison with high-level exercisers. It means that the value of physical activity was not limited in improving cardiovascular function but also can benefit psychological processes or brain health (e.g., delay cognitive decline) (Gregory et al., 2001). Regular fitness made great contribution to decreased mortality and morbidity rates (Gregory et al., 2001; Salmon, 2001; Uffelen et al., 2008). Other evidence also suggested that increased aerobic exercise was associated with structural and functional changes in elders’ brain (Colcombe et al., 2006; Wei et al., 2014). Besides, other crucial sociodemographic factors such as education, literacy, and income also played a vital role in reserving cognitive status. Further evidence about their beneficial impact can be found in numerous aging studies (Perna et al., 2012; Calasanti, 2016; Livingston et al., 2017; Scarmeas et al., 2018).

Above all, as various causes led to cognitive decline in later life, exploring factors in an isolated approach was insufficient to explain cognitive impairment systematically. Instead, it would be appropriate to aggregate multifaceted factors into a unified profile. Only by doing so could clinical guidelines or healthy recommendation be efficient and appropriate, which was also suggested by review work (Plassman et al., 2010) from which a comprehensive consideration of risk and protective factors was necessary when drawing firm conclusions about associations with cognitive decline. The findings in the present study suggested that protective and risk factors were influenced by a number of potentially modifiable variables that could be targets for interventions to promote and reserve better cognitive function. Based on our analysis, the following four types including 12 factors/index would be considered to integrate for predicting cognitive impairment: gender, physical health factors (BMI, chronic disease), socioeconomic and lifestyle factors (education, literacy, Mandarin, marriage, income, and exercise), and psychological health factors (memory, self-care cognition, and anxiety). These potentially modifiable factors (gender excepted) showed promise in preserving cognitive capacity. Targeting these vital factors could help to reduce the incidence of cognitive impairment or substantially delay its onset (Colcombe et al., 2006). It would be constructive to encourage elder people using cognitive stimulation games/activities, like video games, playing cards, language learning, and so on, in which could enormously remedy deficiencies in education or literacy and improve well-being (Charness and Boot, 2009). Meanwhile, physical activity and psychological well-being are also recommended in nursing or clinical practice (McAuley and Rudolph, 1995; Ruuskanen and Ruoppila, 1995; McAuley et al., 2006). Taken together, each factor alone might manifest a spurious and faint association. A comprehensive considering those factors could be valid for predicting cognitive change and preserving cognitive capacity.

Besides, we did not observe significant associations between age and functional impairment. Previous study suggested that age was a major risk factor for cognitive decline (Deary et al., 2009). The reason may be the range of time in the current study was based on only 2 years, not a decade as a previous study (Guralnik et al., 1993). Therefore, the limited segment in our study would be insufficient to reflect the accumulated aging effect. Besides, the effects of smoking and alcohol drinking on cognitive decline were not found to be statistically significant, which was debated in previous evidence (Clare et al., 2017). Evidence was growing that moderate levels of alcohol intake may have a protective effect against dementia and cognitive decline compared with either abstinence or heavy drinking (Ganguli et al., 2005). Moreover, the crude dichotomous classification in our study might conceal the cognitive associations. More precise measurement according to actual consumption was necessary in future studies.

Several other limitations are also needed to be concerned carefully. Firstly, the subjects who participated in the present study came from a remote rural area of China. The external validity of cognitive status reduced and limited its generality. Also, an underestimated cognitive performance may occur for subjects who had relatively lower education and economic status than other older population living in an urban area. Thus, replicating investigation with larger samples and participants living in the city is necessary in future study. Secondly, we did not record information on genetic contribution, brain imaging evidence, healthy dietary habits, and emotional and social support factors, which have previously demonstrated associations with cognitive impairment (Dardiotis et al., 2014). Especially the related social factors, for social organizations are organism-like systems, such as in-group entitativity, which may play as a crucial protective factor for cognitive impairment and improve their life quality in terms of group support and well-being (Campbell, 1958; Pagliaro et al., 2013; Bubbico et al., 2019). Last but not least, we only utilized MMSE and digit span memory test as the measurement of cognitive function which limited the validation and generality of the results we found, though MMSE and digit memory test strongly associated and mutually confirmed the trends of cognitive aging process. Other objective measures, such as electrophysiological and brain imaging technologies, needed to address in future studies. More refined experimental design was also needed to make the protective and risk factors more valid and propel clinical application in cognitive aging field.

## Conclusion

The comprehensive risk and protective effects of sociodemographic, lifestyle, and mental health on cognitive impairment were observed in subjects aged 75 years and older. We found an age-related declining trend of cognitive capacity in each stratified factor with slight diversity associations. The small effect size (R-square: 0.001–0.15) of individual factor suggests that a combined consideration of factors would be appropriate for clinical application and intervention.

## Data Availability Statement

The raw data supporting the conclusions of this article will be made available by the authors, without undue reservation, to any qualified researcher.

## Ethics Statement

The studies involving human participants were reviewed and approved by the Institutional Review Boards of Nanning Normal University. The patients/participants provided their written informed consent to participate in this study.

## Author Contributions

ZY conceived the idea, designed the study, analyzed and interpreted the data, and drafted part of the manuscript. XZ and XH assisted with the analysis and interpretation of data. XZ conducted the experiment. All authors contributed to the article and approved the submitted version.

## Conflict of Interest

The authors declare that the research was conducted in the absence of any commercial or financial relationships that could be construed as a potential conflict of interest.
